# Decompensated metabolic acidosis in the emergency department: Epidemiology, sodium bicarbonate therapy, and clinical outcomes

**DOI:** 10.1016/j.ccrj.2023.05.003

**Published:** 2023-06-24

**Authors:** Christopher Guy, Natasha E. Holmes, Kartik Kishore, Nada Marhoon, Ary Serpa-Neto

**Affiliations:** a Department of Intensive Care, Austin Hospital, Melbourne, Australia; b Data Analytics Research and Evaluation (DARE) Centre, Austin Health and the University of Melbourne, Heidelberg, Victoria, Australia; c Department of Critical Care, School of Medicine, The University of Melbourne, Parkville, Melbourne, Victoria, Australia; d Australian and New Zealand Intensive Care Research Centre, School of Public Health and Preventive Medicine, Monash University, Melbourne, Australia; e Department of Critical Care Medicine, Hospital Israelita Albert Einstein, São Paulo, Brazil

**Keywords:** Emergency department, Acidosis, pH, Base excess, Bicarbonate

## Abstract

**Objective:**

This article aims to describe the epidemiology of decompensated metabolic acidosis, the characteristics of sodium bicarbonate (SB) administration and outcomes in emergency department (ED) patients.

**Design:**

This is a retrospective cohort study.

**Setting:**

ED of a tertiary referral hospital in Melbourne, Australia.

**Participants:**

Adult patients presenting to the ED between 1 July 2011 and 20 September 2020 with decompensated metabolic acidosis diagnosed on arterial blood gas (ABG).

**Main outcome measures:**

We compared characteristics between those treated with or without SB. We studied SB administration characteristics, change in laboratory variables, factors associated with use and dose, and clinical outcomes.

**Results:**

Among 753,613 ED patients, 314 had decompensated metabolic acidosis on ABG, with 17.8% receiving SB. Patients in the SB group had lower median pH, CO2, bicarbonate, and base excess (BE) levels compared with the No SB group (*P* < 0.01). The median number of SB doses in the SB group was one treatment. This was given at a median total dose of 100 mmol and at a median of 2.8 h after the diagnostic blood gas results. Only 42% of patients in the SB group had a subsequent blood gas measured. In such patients, there was no significant change in pH, bicarbonate, or BE. SB therapy was not independently associated with mortality.

**Conclusions:**

ABG-confirmed decompensated metabolic acidosis was rare but associated with a high mortality. SB administration occurred in a minority of patients and in more acidaemic patients. However, SB dose was stereotypical and not tailored to acidosis severity. Assessment of SB effect was infrequent and showed no correction of acidosis. Systematic studies of titrated SB therapy are required to inform current practice.

## 1 Introduction

Acute metabolic acidosis is common in critically ill patients.^1^^,^^2^^,^^3^ It has been associated with worse patient-centred outcomes, including increased hospital length of stay (LOS), increased intensive care unit (ICU) admission, and increased mortality rates.^3^^,^^4^ However, it remains unclear to what extent the acidosis itself or the underlying disease causing the acidosis might be responsible for these outcomes.

Intravenous sodium bicarbonate (SB) is sometimes used in patients with decompensated metabolic acidosis, aiming to mitigate associated negative outcomes. However, previous studies have demonstrated significant practice variation among intensive care clinicians and between different ICUs.^2^^,^^5^ Such observations highlight therapeutic uncertainty, which is in part due to the paucity of large, multicentre randomised controlled trials (RCTs).^6^^,^^7^ The recent BICAR-ICU trial, however, demonstrated a mortality benefit with SB in patients with decompensated metabolic acidosis and acute kidney injury (AKI), spurring renewed interest in SB therapy in ICU patients.^8^

The above data in ICU are not yet mirrored by epidemiological research in emergency department (ED) patients. Yet such work is essential to understand the frequency and current management of decompensated metabolic acidosis at initial hospital presentation. Accordingly, we conducted an epidemiologic study to test the hypothesis that in patients presenting to the ED with arterial blood gas (ABG)-confirmed decompensated metabolic acidosis, SB would be used in a minority of patients and administered at a low dose of approximately 100 mmol as recently observed in the ICU setting.^1^

## 2 Methods

### 2.1 Study design and setting

This was a retrospective cohort study performed at Austin Health, a tertiary referral hospital in Melbourne, Australia. All adult patients ≥18 years of age who presented to the ED between 1 July 2011 and 20 September 2020 with an ABG demonstrating decompensated metabolic acidosis were included. Where patients had multiple ED presentations with metabolic acidosis, only the first was included. The number of patients without an ABG but with a venous blood gas (VBG) suggesting decompensated metabolic acidosis was determined using a modified definition. For the purpose of this study, patients with only VBG assessment were excluded from further analysis. This was required to more accurately identify decompensated metabolic acidosis, as well as to match the ICU literature that currently exists. The Austin Health's Office for Research reviewed this project according to the principles of the National Statement on Ethical Conduct in Research (2007, updated 2018) and approved its conduct. The project number is Audit/19/Austin/74.

### 2.2 Definitions

Decompensated metabolic acidosis was operationally defined as an ABG pH < 7.3 with an ABG base excess (BE) ≤ −4. Where a BE value was not available, an ABG pH < 7.3 with an ABG bicarbonate ≤18 mmol/L and a PaCO2 of ≤40 mmHg (≤5.3 kPa) were used. Where only VBGs were available, a pH < 7.26 was used along with the same BE, bicarbonate, and CO2 criteria.

### 2.3 Data collection

Data extracted from electronic health records included baseline demographics, ICD-10 principal diagnosis codes, all available ABG parameters, full blood examination, and urea and electrolyte panels. While patients were included based on ABG parameters, all VBG data from the patients’ ED admission were also collected. ICD-10 principal diagnosis codes for conditions expected to cause metabolic acidosis were also collected. Diabetic ketoacidosis (DKA), cardiac arrest, and AKI were chosen a priori, while codes relating to infection, sepsis, and septic shock were subsequently collected without further analysis.

The first and last set of ED vital sign data were collected, including respiratory rate (RR), oxygen saturation (SpO2), heart rate (HR), blood pressure (BP), temperature, and conscious state. Finally, the receipt of intravenous SB was recorded and included time-point and quantity of any dose/s given.

### 2.4 Venous blood gas data

While study inclusion criteria required a diagnosis of decompensated metabolic acidosis based on ABG, venous blood gas data for these patients were still collected and utilised in the statistical analysis. This enabled more detailed analysis of blood gas parameter changes and trends in patients over the course of their ED admission.

### 2.5 Statistical analysis

Patients were allocated to either the SB or No SB group depending on SB therapy in the ED. Incidence of decompensated metabolic acidosis and proportion of patients administered SB were calculated. Continuous variables were compared using Wilcoxon rank-sum tests and summarised with median and interquartile range. Categorical variables were compared using Fisher's exact tests and summarised with counts and percentages. All analyses were performed using R-4.0.2 and a *p* < 0.05 was considered statistically significant.

Variables in baseline patient characteristics of each group were compared, including baseline pathology, vital signs, and blood gas parameters. Median number and size of SB doses administered to each patient were calculated. Characteristics of blood gas sampling, including frequency and sample type, were compared between groups. Change in blood gas parameters and vital signs over the course of ED admission were also analysed and compared between the SB and No SB groups.

For the SB group, the median difference between each blood gas parameter measured immediately before and after SB administration was analysed with a mixed-effect median regression model using an internal point algorithm and including patients as random effects to account for the repeated measurements. Furthermore, the change in selected blood gas parameters (median pH, BE, bicarbonate concentration, sodium concentration) was graphically tracked over time for each group and compared using a mixed-effect linear model including time (as a continuous variable), group and an interaction between time × group as fixed effects and patients as random effects to account for the repeated measurements.

Factors associated with the use and total dose of SB were identified using generalised linear models considering a binomial distribution (logistic regression) and generalised linear models. Covariates were included based on a predefined list determined by clinical relevance and further selected if *p* < 0.10 in the univariable model. The effect estimates of continuous variables were based on the effect of increasing each value by one standard deviation. Factors potentially associated with hospital mortality were investigated using the same analysis. Clinical outcomes assessed included ED LOS, ICU admission, ICU LOS, hospital LOS, and hospital mortality.

## 3 Results

### 3.1 Incidence of decompensated metabolic acidosis and SB treatment

**Fig. 1:**
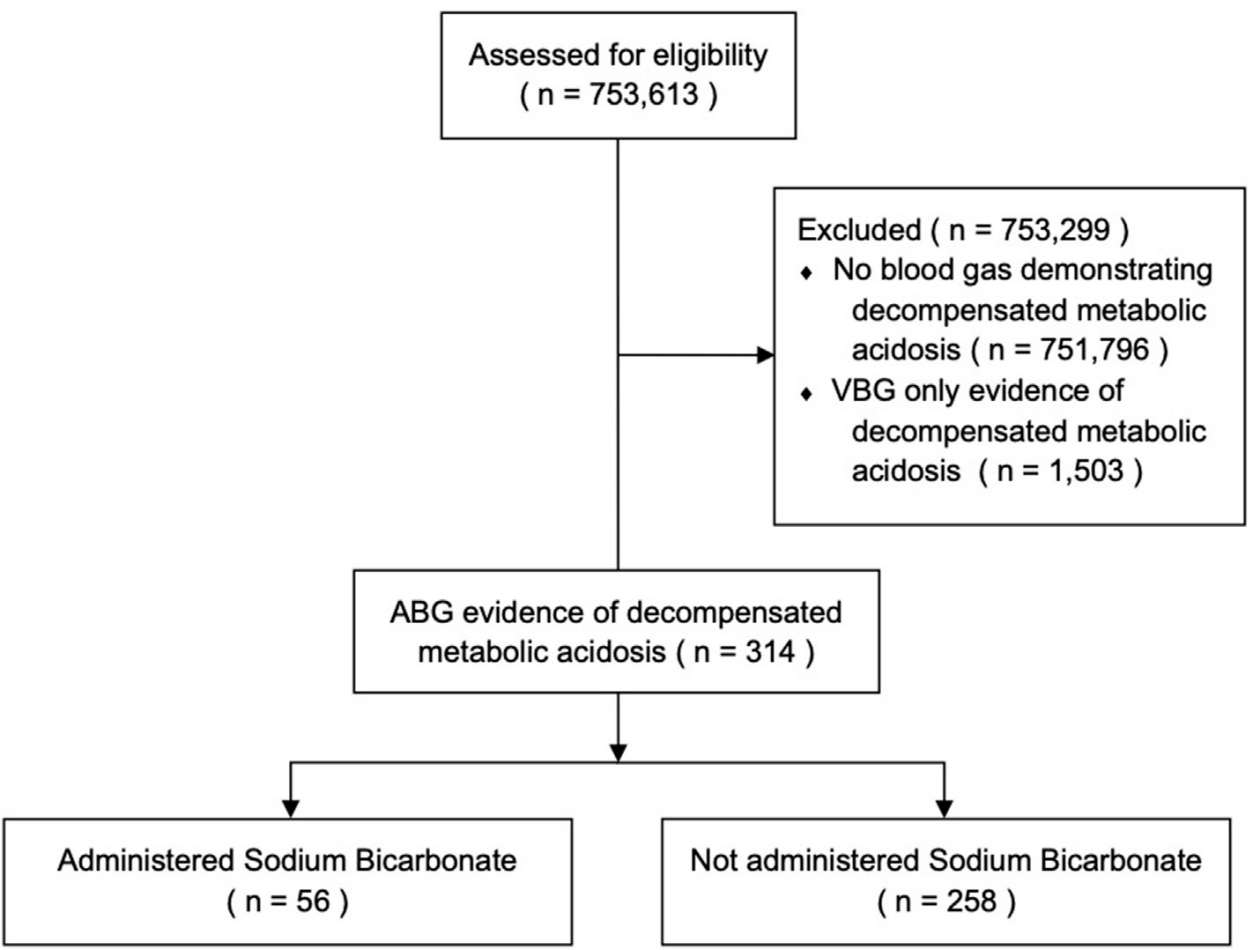
**CONSORT diagram.** Abbreviations: VBG: venous blood gas; ABG: arterial blood gas.

A total of 753,613 patients admitted to the ED were assessed for eligibility over the 3369-day study period. Of these, 314 (0.04%) manifested a decompensated metabolic acidosis on ABG (34 patients per year). Overall, 17.8% (56/314) of these patients received intravenous SB during their ED admission (Fig. 1). A further 1503 patients who only had VBGs manifested a decompensated metabolic acidosis only using the modified VBG-based definition and were excluded from further analysis in this study.

### 3.2 Patient characteristics

Patient characteristics are presented in Table 1. The most common individual ED admission diagnosis was cardiac arrest, followed by DKA and AKI. Patients with an ED admission diagnosis of “other” included those with less common ICD-10 codes. Diagnostic codes relating to infection, sepsis, or septic shock collectively occurred in 56 patients, with 10 receiving SB. Other diagnostic groups included toxicological (14 patients), gastrointestinal bleeding (10 patients), cardiogenic shock (4 patients), and chronic kidney disease (3 patients). Patients in the SB group had lower pH, lower PaCO2, lower bicarbonate, lower BE, and higher potassium levels than patients in the No SB group. The first set of recorded vital signs, however, showed no difference.

**Table 1:** Baseline characteristics of the included patients.

	Overall (*n* = 314)	Bicarbonate (*n* = 56)	No Bicarbonate (*n* = 258)	*p* value
Age, years	67 (50–80)	67 (56–80)	67 (49–80)	0.532
Male gender - no. (%)	180 (57.3)	28 (50.0)	152 (58.9)	0.236
ED admission diagnosis - no. (%)				0.063
Acute kidney injury	23 (7.3)	9 (16.1)	14 (5.4)	
Cardiac arrest	30 (9.6)	6 (10.7)	24 (9.3)	
Diabetic ketoacidosis	36 (11.5)	5 (8.9)	31 (12.0)	
Other	225 (71.7)	36 (64.3)	189 (73.3)	
Baseline pathology				
Creatinine, μmol/L	82 (63–125)	85 (62–106)	82 (63–130)	0.777
Platelet, x 10^9^/L	239 (184–299)	246 (195–295)	236 (184–299)	0.658
Haemoglobin, g/L	125 (102–139)	129 (111–140)	124 (100–138)	0.157
White blood cell count, 10^9^/L	8.1 (6.2–11.2)	8.6 (5.9–11.6)	8.0 (6.3–11.1)	0.893
First vital signs				
Heart rate, bpm	100 (80–120)	92 (78–113)	100 (80–120)	0.146
Systolic blood pressure, mmHg	115 (93–136)	110 (93–140)	115 (94–135)	0.977
Diastolic blood pressure, mmHg	68 (55–80)	68 (53–80)	68.5 (55–80)	0.516
Respiratory rate, breaths/min	22 (18–29)	22 (18–28)	22 (18–30)	0.690
SpO_2_, %	98 (94–100)	98 (96–100)	98 (94–100)	0.024
FiO_2_	0.21 (0.21–0.51)	0.21 (0.21–0.50)	0.21 (0.21–0.52)	0.403
SpO_2_/FiO_2_ ratio	429 (182–467)	467 (196–471)	419 (174–467)	0.080
Temperature, ºC	35.9 (35.2–36.6)	35.7 (34.4–36.4)	35.9 (35.2–36.6)	0.212
First blood gas				
Sample - no. (%)				0.064
Arterial	173 (55.1)	23 (41.1)	150 (58.1)	
Venous	124 (39.5)	31 (55.4)	93 (36.0)	
Unknown	17 (5.4)	2 (3.6)	15 (5.8)	
pH	7.18 (7.06–7.25)	7.09 (6.96–7.19)	7.19 (7.08–7.26)	<0.001
PaCO_2_, mmHg	35 (28–40)	31 (25–37)	36 (28–43)	0.001
Bicarbonate, mmol/L	13 (8–17)	10 (6–12)	14 (9–17)	<0.001
Base excess, mmol/L	−10.2 (−14.8–−6.6)	−16.5 (−19.4–−10.6)	−9.7 (−14.2–−6.3)	0.026
Sodium, mmol/L	135 (130–139)	135 (129–139)	135 (131–139)	0.455
Potassium, mmol/L	4.7 (3.9–5.7)	5.3 (4.1–6.3)	4.6 (3.9–5.5)	0.016
Chloride, mmol/L	106 (101–110)	104 (98–112)	106 (101–110)	0.879

Data are median (quartile 25th - quartile 75th) or N (%).

Abbreviations: ED: emergency department; ICU: intensive care unit.

### 3.3 Blood gas sampling, sodium bicarbonate use, and change in blood gas parameters

As shown in Table 2, the SB group had more blood gas measurements than the No SB group. The median number of bicarbonate doses administered to patients in the SB group was 1, with a median total dose of 100 mmol. SB was administered a median of 2.8 h after the preceding blood gas result (Table 3). A subsequent blood gas was measured a median of 0.6 h following commencement of SB; however, this was only performed in 23 patients (42%) in the SB group.

**Table 2:** Bicarbonate administration and blood gases.

	Overall (*n* = 314)	Bicarbonate (*n* = 56)	No Bicarbonate (*n* = 258)	*p* value
Number of bicarbonate doses	1 (1–2)	1 (1–2)	–	–
Total dose, mmol	100 (100–200)	100 (100–200)	–	–
Range, mmol	25–1000	25–1000		
Mode, mmol	100	100		
Hours until the first dose	3.9 (2.1–6.9)	3.9 (2.1–6.9)	–	–
Total number of blood gases	2 (1–3)	3 (2–4)	2 (1–3)	0.026
Last blood gas in ED				
Sample - no. (%)				0.334
Arterial	244 (86.8)	18 (78.3)	226 (87.6)	
Venous	32 (11.4)	5 (21.7)	27 (10.5)	
Unknown	5 (1.8)	0 (0.0)	5 (1.9)	
pH	7.22 (7.13–7.27)	7.08 (6.97–7.20)	7.23 (7.14–7.27)	<0.001
PaCO_2_, mmHg	35 (27–40)	28 (22–35)	35 (28–40)	0.003
Bicarbonate, mmol/L	14 (9–17)	8 (5–11)	14 (10–17)	<0.001
Base excess, mmol/L	−13.0 (−18.9–−9.7)	−22.2 (−26.4–−17.3)	−12.7 (−17.8–−9.6)	<0.001
Sodium, mmol/L	136 (132–139)	137 (134–142)	136 (132–139)	0.107
Potassium, mmol/L	4.5 (3.9–5.2)	5.0 (4.2–5.8)	4.4 (3.9–5.2)	0.071
Chloride, mmol/L	107 (102–111)	103 (100–112)	107 (102–111)	0.334
Change in blood gas^a^				
pH	0.00 (0.00–0.08)	0.05 (0.00–0.17)	0.00 (0.00–0.07)	0.056
PaCO_2_, mmHg	0.00 (−4.00–0.00)	0.00 (−1.00–3.00)	0.00 (−4.00–0.00)	0.154
Bicarbonate, mmol/L	0.00 (0.00–2.00)	1.00 (0.00–3.00)	0.00 (0.00–1.00)	0.057
Base excess, mmol/L	0.00 (−0.40–2.43)	−4.50 (−4.50–−4.50)	0.00 (−0.35–2.65)	0.127
Sodium, mmol/L	0.00 (0.00–1.00)	3.00 (1.00–6.00)	0.00 (−1.00–1.00)	<0.001
Potassium, mmol/L	0.00 (−0.40–0.00)	−0.90 (−1.38–−0.30)	0.00 (−0.40–0.00)	<0.001
Chloride, mmol/L	0.00 (0.00–2.00)	0.00 (−1.00–2.00)	0.00 (0.00–2.00)	0.531

Data are median (quartile 25th - quartile 75th) or N (%).

a Last - first blood gas.

**Table 3:** Blood gas immediately before and after bicarbonate administration.

	Before (*n* = 53)	After (*n* = 23)	Median Difference (95% CI)	*p* value
Hours between blood gas and bicarbonate use	−2.8 (−4.0–−1.3)	0.6 (0.3–1.2)	3.45 (2.67–4.23)	<0.001
Sample				0.020
Arterial	20 (37.7)	16 (69.6)		
Venous	31 (58.5)	6 (26.1)		
Unknown	2 (3.8)	1 (4.3)		
pH	7.09 (6.94–7.19)	7.07 (7.00–7.17)	−0.02 (−0.09 to 0.05)	0.597
Base excess, mmol/L	−20.0 (−26.6–−16.3)	−22.2 (−26.5–−16.8)	−2.20 (−6.07 to 1.66)	0.267
PaCO_2_, mmHg	31.0 (25.0–35.0)	30.0 (21.0–35.0)	−1.00 (−7.51 to 5.51)	0.764
Bicarbonate, mmol/L	9.0 (6.0–12.0)	8.0 (5.0–11.5)	−1.00 (−3.90 to 1.90)	0.502
Sodium, mmol/L	134.0 (129.0–139.0)	137.0 (133.5–141.5)	3.00 (−0.67 to 6.67)	0.114
Potassium, mmol/L	5.2 (4.1–6.6)	5.2 (4.0–5.8)	−0.04 (−0.93 to 0.85)	0.930
Chloride, mmol/L	104.0 (98.0–111.5)	103.0 (98.5–112.5)	−1.00 (−6.87 to 4.87)	0.739
Ionised calcium	1.2 (1.1–1.3)	1.2 (1.0–1.3)	0.02 (−0.12 to 0.15)	0.822
Haemoglobin, g/L	108.0 (92.0–126.8)	89.0 (77.8–108.2)	−17.86 (−31.91 to −3.80)	0.015
Glucose, mmol/L	8.7 (6.7–14.7)	14.8 (7.5–24.1)	6.28 (−0.01 to 12.58)	0.054
Lactate, mmol/L	7.8 (2.3–13.6)	8.3 (1.9–17.5)	0.50 (−6.79 to 7.79)	0.893

Data are median (quartile 25th - quartile 75th) or N (%).

The median bicarbonate concentration decreased over the course of ED admission in the SB group, while it remained stable in the No SB group (Table 2). This difference between groups reached statistical significance (*p* < 0.001). There was no significant difference when comparing groups for changes in pH, PaCO2, or BE.

Table 3 shows the change in blood gas parameters when comparing values immediately before and after SB administration. Of the 56 patients treated with SB, 53 had at least one blood gas measurement before intervention. In 44 patients, this included at least one ABG, while 9 patients only had VBGs before treatment but an ABG thereafter. There was no change in blood gas parameters, except for a reduction in haemoglobin from 108 g/L to 89 g/L (*p* = 0.015). Median blood pH was 7.09 before SB administration and 7.07 when remeasured in a subsequent blood gas sample. Median bicarbonate concentration decreased from 9 to 8 mmol/L and BE from −20 to −22.2 mEq/L.

**Fig. 2:**
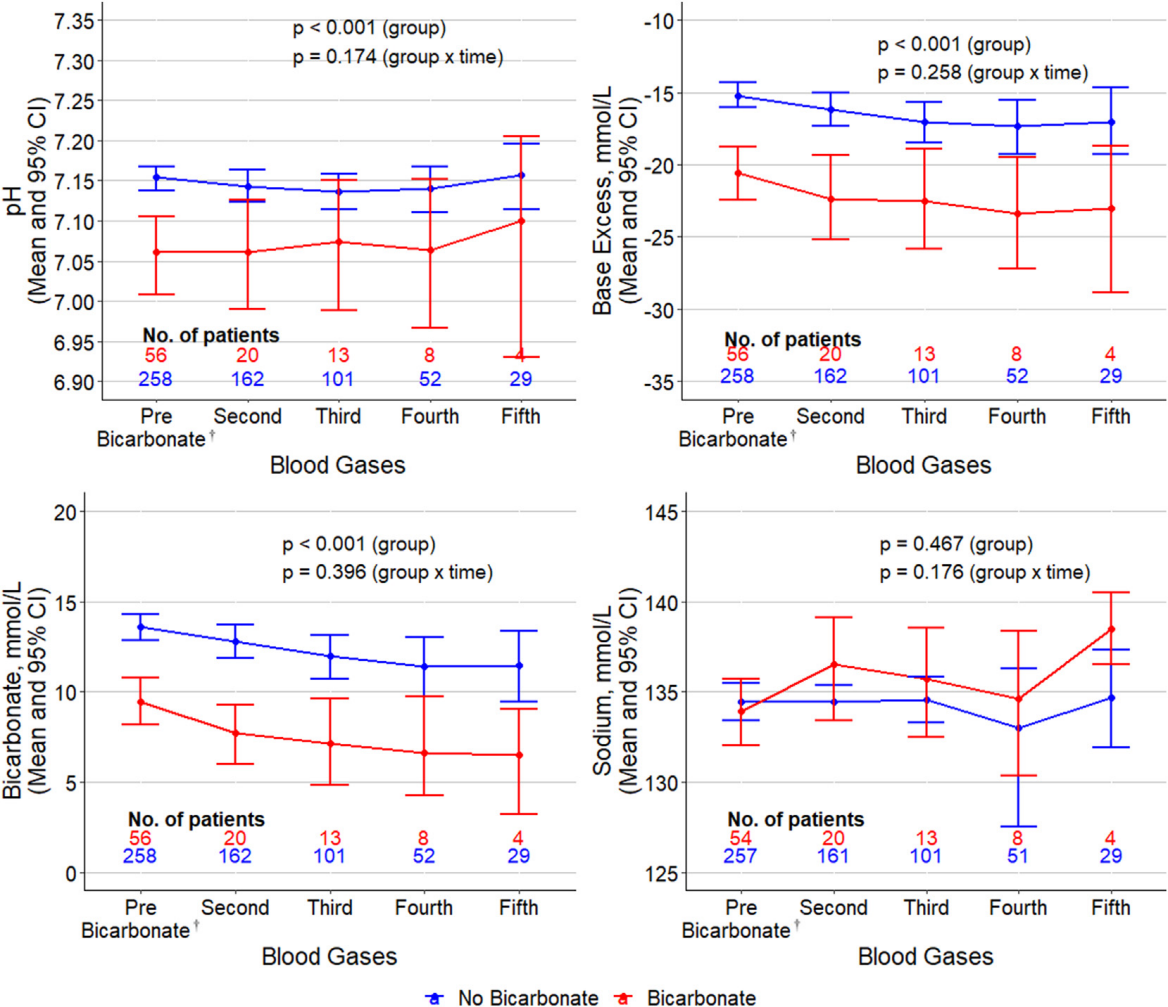
**Change in blood gas parameters over course of ED admission for SB and No SB groups**. While baseline blood gas pH, BE, and bicarbonate were significantly different at baseline between groups, there was no significant change in this difference measured in blood gases following SB administration. Blood gas sodium concentration was not significantly different between groups over the course of ED admission. † The initial non-SB blood gas represents the first value obtained in the ED.

Fig. 2 illustrates that while baseline blood gas pH, BE, and bicarbonate levels were significantly different at baseline between groups, there was no significant change in this difference following SB administration.

### 3.4 SB administration and vital signs

Table S1 shows the association of SB administration with vital signs. The median RR in the SB group did not change, while it decreased by 2 breaths per minute in the No SB group (*p* = 0.021). Changes in other vital signs were not associated with SB administration.

### 3.5 Factors associated with the use and total dose of SB

On univariable analysis, AKI diagnosis and higher potassium levels were associated with a higher chance of SB administration (Table S2). Higher pH, PaCO2, bicarbonate, and BE levels were associated with a lower chance of SB administration. No clinical parameter at ED admission was associated with SB administration. After adjustment for confounders, however, the diagnosis of DKA and a higher bicarbonate level were associated with a lower chance of SB administration (Table S2). Factors associated with SB dose are shown in Table S3 and show that a lower pH was the dominant factor associated with the amount of SB administered.

### 3.6 Clinical outcomes and factors associated with hospital mortality

As shown in Table S4, hospital mortality occurred in 22.9% of the overall study population. The SB group experienced a higher unadjusted rate of hospital mortality compared with the No SB group. However, ED LOS showed no significant difference between the SB and No SB groups. Overall, 75.2% of patients required ICU admission, also with no significant difference between the SB and No SB groups. Similarly, both ICU and hospital LOS were not significantly different between groups. After adjustment, the only factor independently associated with hospital mortality was an ED admission diagnosis of cardiac arrest (Table 4). Use of SB was not independently associated with hospital mortality.

**Table 4:** Relationship of sodium bicarbonate therapy with hospital mortality adjusted for first blood gas results.

	Odds Ratio (95% CI)	*p* value
Use of bicarbonate	2.43 (0.43–11.86)	0.278
ED admission diagnosis		
Other	1 (Reference)	
Acute kidney injury	NA	–
Cardiac arrest	4.02 (1.00–15.99)	0.045
Diabetic ketoacidosis	NA	–
First blood gas		
pH	3.98 (0.23–122.88)	0.371
PaCO_2_	1.44 (0.22–10.96)	0.709
Bicarbonate	2.28 (0.03–131.40)	0.692
Base excess	0.18 (0.00–13.44)	0.411

Effect estimates of continuous variables represent the effect of the increase of one standard deviation.

pH and base excess had multicollinearity with bicarbonate. CI, confidence interval. NA, not applicable, indicates the sample size was insufficient to calculate the effect estimate.

## 4 Discussion

### 4.1 Key findings

We conducted a retrospective cohort study of the incidence of ABG-confirmed decompensated metabolic acidosis and the administration of intravenous SB in a tertiary ED over close to a decade. ABG-confirmed decompensated metabolic acidosis was rare but associated with a high rate of hospital mortality. SB was administered to one in six of these patients, in association with a lower initial blood pH, bicarbonate concentration, and BE. Its dose was most commonly 100 mmol (typical single bottle) and given a median of 2.8 h after the preceding blood gas result. Furthermore, only 50% of patients had a repeat blood gas following bicarbonate administration. In those patients who did have a repeat blood gas, there was no change in pH or bicarbonate.

### 4.2 Relationship to previous literature in the ED

Epidemiological data describing decompensated metabolic acidosis in the ED is scarce. Previous studies have focused on DKA or trauma. Similarly, outcome data for patients with metabolic acidosis in the ED have been limited to specific settings, including out-of-hospital cardiac arrest (OOHCA),[9], [10], [11] acute decompensated heart failure,^12^^,^^13^ poisonings,^14^ sepsis,^15^^,^^16^ traumatic brain injury,^17^ and trauma.^18^ Lactataemia with or without acidosis has also been investigated for any prognostic utility in ED patients.[19], [20], [21], [22], [23], [24], [25]

Similarly, the literature regarding SB therapy in the ED primarily relates to DKA.^26^^,^^27^ We undertook a literature review on the use of SB for metabolic acidosis in the ED, excluding DKA. We screened 8613 articles, with full-text assessment of 93 and identified only two relevant studies, both of which focused on cardiac arrest patients.^28^^,^^29^

The first study was a retrospective, matched case–control analysis of 599 OOHCA patients to evaluate the association between SB administration and chance of achieving return of spontaneous circulation (ROSC).^28^ Patients were included if they had not achieved prehospital ROSC and therefore required advanced cardiac life support in the ED. SB administration and its total dose were both associated with occurrence of ROSC within 20 min.^28^ The second study was an RCT of SB versus placebo in 50 patients admitted to the ED with nontraumatic OOHCA who had not achieved ROSC after 10 min and had a severe metabolic acidosis.^29^ The administration of a single 50 mmol bolus of SB did not have an impact on rates of ROSC or neurological outcome at 1 month. There was an increase in bicarbonate level but no significant change in pH.^29^

To mirror ED data, the epidemiology of decompensated metabolic acidosis in the ICU setting was recently investigated in an international retrospective observational study.^1^ The administration of SB occurred in 18% of such patients with a median total dose of 110 mmol, similar to the 100 mmol administered in our ED population.^1^ The dosage of SB did not correlate with body weight, BE, or bicarbonate levels, and SB administration was not independently associated with ICU or hospital mortality.^1^

Finally, there are no studies of the epidemiology and treatment of metabolic acidosis diagnosed and managed with venous blood gases.

### 4.3 Implications of study findings

Our findings imply that ABG-confirmed decompensated metabolic acidosis is infrequent in ED patients but associated with a high mortality. By showing that one in six patients received SB, they also imply that clinicians chose to administer such treatment in a minority of patients. Nonetheless, the details of dosing also imply stereotypical prescription in most cases (one bottle) with no adjustment for severity, patient size, and little monitoring of effect. Furthermore, SB was administered a median of 2.8 h after the diagnosis of decompensated metabolic acidosis. In their aggregate, these findings suggest an opportunity for the exploration of titrated and rapid therapy in this setting.

### 4.4 Study strengths and limitations

Our study provides the first description of the epidemiology of decompensated metabolic acidosis and the characteristics of SB administration in the ED setting. Our findings are based on a comprehensive dataset including 314 patients identified from 753,613 patients admitted to a tertiary ED. This provided novel information on the incidence and outcomes of patients diagnosed with this condition. Furthermore, by describing the practice of administering SB within this group, including dose, patient characteristics, changes in blood gas and clinical parameters, and association with clinical outcomes, our study provides essential information to justify, design, and power future RCTs.

We acknowledge several limitations. This is an observational study, and therefore, causal inferences are not possible. A retrospective study design was required due to the low frequency of decompensated metabolic acidosis in the ED. However, most data were electronically recorded and collected, mitigating any risk of selection bias. This was a single-centre study; however, during the study period, the ED admitted over 750,000 patients and treatment was delivered by an estimated cohort of rotating senior clinicians, trainees, and registrars numbering >100. The ABG-based inclusion criteria meant that any patients administered SB who only had VBGs would have been excluded. This was required to more accurately identify decompensated metabolic acidosis as well as to match the ICU literature that currently exists. However, our findings indicate that the number of patients with decompensated metabolic acidosis as diagnosed by VBG may be fivefold greater. The characteristics, outcomes, and management of such patients require separate investigation.

Furthermore, patients without any blood gas result in the ED could also have undiagnosed decompensated metabolic acidosis and therefore would also not be represented in this study. Finally, data regarding vasopressor use or the use of mechanical ventilation in the ED for these patients was not included in the dataset, and this will have some effect on the interpretation of clinical parameters.

## 5 Conclusion

In the ED of a tertiary Australian hospital over a period of almost a decade, ABG-confirmed decompensated metabolic acidosis was rare but associated with a high mortality. SB was administered to approximately one in six patients and was more common in the presence of severe acidaemia. The administered dose, however, was late and stereotypical, reflecting the size of the available bottle rather than the size of the patient or the severity of the acidosis. Assessment of the effect of SB was infrequent and, when performed, showed no correction of the pathophysiological derangement. These findings provide a rationale for systematic studies of early and titrated SB therapy.

## Credit author statement

**Christopher Guy:** Conceptualisation, Methodology, Data Curation, Writing – Original Draft, Reviewing and Editing. **Natasha E Holmes:** Conceptualisation, Data Curation, Writing – Reviewing and Editing. **Kartik Kishore:** Data Curation. **Nada Marhoon:** Data Curation. **Ary Serpa-Neto:** Methodology, Formal analysis.

## Conflict of interest

The authors declare they have no conflict of interest.
