# Supplementary material for: Decompensated metabolic acidosis in the emergency department: Epidemiology, sodium bicarbonate therapy, and clinical outcomes
Source: Crit Care Resusc. 2023 Jun 24;25(2):71–7. doi: 10.1016/j.ccrj.2023.05.003 (PMC10581257; doi:10.1016/j.ccrj.2023.05.003)
Supplement: Multimedia component 1 [file mmc1.doc]

| **Table S1 - Vital Signs Over the ED Stay** | | | | |
| --- | --- | --- | --- | --- |
|  | **Overall**  **(*n* = 314)** | **Bicarbonate**  **(*n* = 56)** | **No Bicarbonate**  **(*n* = 258)** | ***p* value** |
| First vital signs |  |  |  |  |
| Heart rate, bpm | 100.0 (80.0 - 120.0) | 92.5 (78.2 - 112.8) | 100.0 (80.0 - 120.0) | 0.146 |
| Systolic blood pressure, mmHg | 115.0 (93.2 - 136.0) | 110.0 (93.5 - 140.0) | 115.0 (93.8 - 135.2) | 0.977 |
| Diastolic blood pressure, mmHg | 68.0 (55.0 - 80.0) | 68.0 (53.2 - 80.0) | 68.5 (55.0 - 80.0) | 0.516 |
| Respiratory rate, breaths/min | 22.0 (18.0 - 29.2) | 22.0 (18.0 - 28.0) | 22.0 (18.0 - 30.0) | 0.690 |
| SpO_2_, % | 98.0 (94.0 - 100.0) | 98.0 (96.2 - 100.0) | 98.0 (94.0 - 100.0) | 0.024 |
| FiO_2_ | 0.21 (0.21 - 0.51) | 0.21 (0.21 - 0.50) | 0.21 (0.21 - 0.52) | 0.403 |
| SpO_2_ / FiO_2_ ratio | 428.6 (181.8 - 466.7) | 466.7 (196.0 - 471.4) | 419.0 (174.5 - 466.7) | 0.080 |
| Temperature, ºC | 35.9 (35.2 - 36.6) | 35.7 (34.4 - 36.4) | 35.9 (35.2 - 36.6) | 0.212 |
| Last vital signs |  |  |  |  |
| Heart rate, bpm | 96.0 (80.0 - 112.0) | 96.5 (85.0 - 109.2) | 96.0 (79.0 - 112.8) | 0.844 |
| Systolic blood pressure, mmHg | 109.0 (97.0 - 131.0) | 106.5 (91.2 - 130.8) | 110.5 (97.0 - 131.0) | 0.443 |
| Diastolic blood pressure, mmHg | 62.0 (50.0 - 74.0) | 59.0 (49.2 - 68.0) | 63.5 (50.0 - 75.0) | 0.057 |
| Respiratory rate, breaths/min | 20.0 (16.0 - 25.2) | 22.0 (18.5 - 27.5) | 20.0 (16.0 - 24.0) | 0.018 |
| SpO_2_, % | 99.0 (96.0 - 100.0) | 99.0 (96.0 - 100.0) | 98.0 (96.0 - 100.0) | 0.326 |
| FiO_2_ | 0.32 (0.21 - 0.55) | 0.21 (0.21 - 0.50) | 0.35 (0.21 - 0.55) | 0.233 |
| SpO_2_ / FiO_2_ ratio | 305.1 (178.1 - 466.7) | 381.0 (192.0 - 471.4) | 285.7 (175.5 - 466.7) | 0.207 |
| Temperature, ºC | 35.9 (35.3 - 36.5) | 35.7 (34.8 - 36.6) | 35.9 (35.3 - 36.5) | 0.200 |
| Change in vital signs† |  |  |  |  |
| Heart rate, bpm | -0.50 (-13.00 - 5.00) | 0.00 (-6.00 - 5.00) | -2.00 (-14.00 - 4.00) | 0.037 |
| Systolic blood pressure, mmHg | 0.00 (-16.75 - 11.00) | 0.00 (-21.75 - 12.50) | 0.00 (-16.00 - 11.00) | 0.789 |
| Diastolic blood pressure, mmHg | -2.00 (-17.00 - 5.00) | -3.50 (-19.50 - 6.75) | -2.00 (-16.00 - 4.75) | 0.588 |
| Respiratory rate, breaths/min | -1.00 (-5.25 - 2.00) | 0.00 (-2.50 - 4.00) | -2.00 (-6.00 - 1.00) | 0.021 |
| SpO_2_, % | 0.00 (-1.00 - 3.00) | 0.00 (-1.75 - 2.00) | 0.00 (-1.00 - 3.00) | 0.112 |
| FiO_2_ | 0.00 (0.00 - 0.07) | 0.00 (0.00 - 0.00) | 0.00 (0.00 - 0.09) | 0.439 |
| SpO_2_ / FiO_2_ ratio | 0.00 (-71.14 - 10.27) | 0.00 (-14.29 - 4.76) | 0.00 (-80.86 - 19.00) | 0.907 |
| Temperature, ºC | 0.00 (-0.40 - 0.57) | 0.00 (-0.60 - 0.55) | 0.00 (-0.40 - 0.57) | 0.806 |
| Data are median (quartile 25^th^ - quartile 75^th^) or N (%).  †Last - first value. | | | | |

| **Table S2 - Factors Associated with the Use of Bicarbonate** | | | | |
| --- | --- | --- | --- | --- |
|  | **Univariable** | | **Multivariable**† | |
|  | **Odds Ratio**  **(95% CI)** | ***p* value** | **Odds Ratio**  **(95% CI)** | ***p* value** |
| Age | 1.15 (0.86 to 1.56) | 0.362 | --- | --- |
| Male gender | 0.70 (0.39 to 1.25) | 0.223 | --- | --- |
| ED admission diagnosis |  |  |  |  |
| Other | 1 (Reference) |  | 1 (Reference) |  |
| Acute kidney injury | 3.37 (1.32 to 8.30) | 0.009 | 1.89 (0.66 to 5.12) | 0.215 |
| Cardiac arrest | 1.31 (0.46 to 3.26) | 0.580 | 1.12 (0.35 to 3.17) | 0.843 |
| Diabetic ketoacidosis | 0.85 (0.27 to 2.16) | 0.747 | 0.30 (0.09 to 0.83) | 0.031 |
| Baseline creatinine | 0.80 (0.54 to 1.10) | 0.210 | --- | --- |
| Baseline WCC | 0.94 (0.67 to 1.25) | 0.699 | --- | --- |
| Presenting vital signs |  |  |  |  |
| Heart rate | 0.85 (0.62 to 1.14) | 0.284 | --- | --- |
| Systolic blood pressure | 0.98 (0.73 to 1.32) | 0.911 | --- | --- |
| Respiratory rate | 0.92 (0.68 to 1.23) | 0.596 | --- | --- |
| FiO_2_ | 0.96 (0.66 to 1.36) | 0.844 | --- | --- |
| Temperature | 0.82 (0.61 to 1.11) | 0.184 | --- | --- |
| First blood gas |  |  |  |  |
| pH | 0.55 (0.41 to 0.72) | < 0.001 | --- | --- |
| PaCO_2_ | 0.56 (0.37 to 0.81) | 0.004 | 1.48 (0.78 to 2.65) | 0.200 |
| Bicarbonate | 0.45 (0.31 to 0.63) | < 0.001 | 0.30 (0.16 to 0.54) | < 0.001 |
| Base excess | 0.53 (0.29 to 0.93) | 0.027 | --- | --- |
| Sodium | 0.94 (0.72 to 1.27) | 0.656 | --- | --- |
| Potassium | 1.32 (1.01 to 1.73) | 0.042 | 1.14 (0.83 to 1.54) | 0.408 |
| Chloride | 1.01 (0.76 to 1.38) | 0.969 | --- | --- |
| Effect estimates of continuous variables represent the effect of the increase of one standard deviation.  †Including variables with *p* < 0.100 in the univariable model. pH and base excess were excluded due to multicollinearity with bicarbonate. | | | | |

| **Table S3 - Factors Associated with the Total Dose of Bicarbonate Used** | | | | |
| --- | --- | --- | --- | --- |
|  | **Univariable** | | **Multivariable**† | |
|  | **Mean Difference**  **(95% CI)** | ***p* value** | **Mean Difference**  **(95% CI)** | ***p* value** |
| Age | -33.40 (-77.80 to 11.00) | 0.137 | --- | --- |
| Male gender | -50.89 (-129.21 to 27.43) | 0.198 | --- | --- |
| ED admission diagnosis |  |  |  |  |
| Other | 1 (Reference) |  |  |  |
| Acute kidney injury | -17.36 (-130.38 to 95.66) | 0.759 | --- | --- |
| Cardiac arrest | -6.25 (-139.98 to 127.48) | 0.926 | --- | --- |
| DKA | -2.92 (-147.66 to 141.82) | 0.968 | --- | --- |
| Baseline creatinine | 1.49 (-57.20 to 60.17) | 0.960 | --- | --- |
| Baseline WCC | -43.91 (-101.16 to 13.34) | 0.130 | --- | --- |
| Presenting vital signs |  |  |  |  |
| Heart rate | -1.04 (-40.30 to 38.21) | 0.958 | --- | --- |
| Systolic blood pressure | 40.12 (0.46 to 79.77) | 0.047 | 38.67 (2.93 to 74.42) | 0.035 |
| Respiratory rate | -19.65 (-60.56 to 21.25) | 0.340 | --- | --- |
| FiO_2_ | 41.33 (-9.71 to 92.38) | 0.109 | --- | --- |
| Temperature | -22.23 (-64.56 to 20.10) | 0.296 | --- | --- |
| First blood gas |  |  |  |  |
| pH | -52.09 (-79.78 to -24.39) | < 0.001 | -54.54 (-81.62 to -27.47) | < 0.001 |
| PaCO_2_ | -8.50 (-62.32 to 45.31) | 0.753 | --- | --- |
| Bicarbonate | -37.87 (-84.04 to 8.30) | 0.106 | --- | --- |
| Base excess | -32.41 (-77.81 to 12.98) | 0.141 | --- | --- |
| Sodium | 45.25 (-0.58 to 91.08) | 0.053 | 33.05 (-7.88 to 73.98) | 0.111 |
| Potassium | 7.15 (-34.49 to 48.78) | 0.732 | --- | --- |
| Chloride | 28.38 (-10.55 to 67.31) | 0.150 | --- | --- |
| Effect estimates of continuous variables represent the effect of the increase of one standard deviation.  †Including variables with *p* < 0.100 in the univariable model. | | | | |

| **Table S4 - Clinical Outcomes without adjustment for baseline imbalances** | | | | |
| --- | --- | --- | --- | --- |
|  | **Overall**  **(*n* = 314)** | **Bicarbonate**  **(*n* = 56)** | **No Bicarbonate**  **(*n* = 258)** | ***p* value** |
| ED length of stay, hours | 5.3 (3.5 - 8.3) | 5.2 (3.4 - 9.0) | 5.4 (3.6 - 8.3) | 0.893 |
| ICU admission - no. (%) | 215 / 286 (75.2) | 38 / 50 (76.0) | 177 / 236 (75.0) | 0.999 |
| ICU length of stay, hours | 65.0 (29.0 - 162.0) | 47.5 (31.0 - 155.8) | 66.0 (29.0 - 160.0) | 0.625 |
| Hospital length of stay, days | 6.5 (2.2 - 15.6) | 5.6 (1.7 - 17.5) | 6.6 (2.3 - 15.0) | 0.805 |
| Hospital mortality - no. (%) | 72 (22.9) | 20 (35.7) | 52 (20.2) | 0.021 |
| Data are median (quartile 25^th^ - quartile 75^th^) or N (%).  Abbreviations: ED: emergency department; ICU: intensive care unit. | | | | |
